# The Guy's Appeal

**Published:** 1920-07-31

**Authors:** 


### The Hospital

The Workers' Newspaper of Administrative Medicine and Institutional Life, Administration, National Insurance and Health.

No. 1782, Vol. LXVIII. SATURDAY, JULY 31, 1920. PRICE TWOPENCE

### THE GUY'S APPEAL.

The annual report of Guy's Hospital which has been recently published makes two special points of appeal to the public generosity and the people's sympathy with the voluntary hospital system. We well know the state of perfection as a modern, well-equipped hospital to which Guy's has of late years been brought, owing in no small measure to this tireless energy of the late superintendent, Sir E. Cooper Perry, and we have time and again expressed our appreciation of the valuable part which Guy's and its staff have played in the affairs of a progressive hospital world.

Now we see Guy's publishing an appeal at a time when the question of raising funds for the voluntary hospitals is a topic of widespread interest, and apart from our first-hand knowledge of how richly a whole-hearted public response is deserved, we are glad to note that the particular ideals and immediate needs which Viscount Goschen brings forward are in such substantial agreement with those we have repeatedly urged as being of paramount importance both to the actual administration of a voluntary hospital and to the well-being of the public itself.

In the first place, it is pointed out that Guy's, which benefits nearly 110,000 in- and out-patients annually, with an expenditure of £130,000 per annum, has been hitherto conducted with a yearly subscription list of under £3,500. As with every institution at the present time, the enormous advance in prices of food, bedding, surgical dressings, fuel, labour, etc., since the war has greatly increased the cost of maintenance and administration. We see from the King's Fund statistics that, large though these increases are,—in 1913 a bed in constant occupation at Guy's cost £87 17s. 1d. a year, while in 1918 the corresponding figure was £131 12s. 6d., and will be higher still in 1920,—yet, as far as Guy's is concerned, they are much below the average for the group of large hospitals in London having medical schools attached to them. In any case there is no doubt that for administrative excellence Guy's has deserved, and does still deserve, much credit and appreciation. At the moment, however, although the governors have devised, and the King's Fund has approved, large schemes of capital outlay, aggregating in all about £200,000, yet they express their appreciation of the fact that very many other hospitals are urging much-needed extension schemes upon the public notice, and accordingly Guy's is at present seeking rather to attract the annual subscription and the donation for the purpose of maintenance than to obtain large sums for increasing the number of their beds. From this point of view it should be noted that the assured income of Guy's from landed property, from its Re-endowment Fund, and from all other sources is estimated at £80,000 per annum, while, as stated above, against this there is the inevitable expenditure of £130,000 per annum if the whole in- and out-patient service is to be kept up. This potential annual deficiency of £50,000 must be made good. Guy's appeals to the public particularly for a further development of that all-important source, the small annual subscription.

The second section applies to the *endowment* of medical education and research. The value directly and indirectly to the general public of these sections of a hospital's work have of late received great and deserved emphasis, and whether for Guy's or for the medical schools in general it is unnecessary here to repeat our enthusiasm and appreciation of the enormous value of this form of public support. The debt upon the present excellent school buildings at Guy's is still considerable, and also it is to be remembered that the Guy's Medical School has hitherto been carried on entirely without endowment, a circumstance only possible owing to great and willing sacrifices by all members of the medical staff concerned.

